# New genetic variants of *Toxoplasma gondii* isolates and a novel *ROP5* allele in free-range chickens from Tabasco, México

**DOI:** 10.1017/S0031182025100401

**Published:** 2025-09

**Authors:** Luis Fernando Valenzuela-Moreno, Carlos Cedillo-Peláez, Claudia Patricia Rico-Torres, Lluvia Guadalupe Moreno-Pérez, Claudia Virginia Zaragoza-Vera, Luz Belinda Ortiz-Alegría, Irma Cañedo-Solares, Lizbeth Xicoténcatl-García, Fernando García-Lacy, Heriberto Caballero-Ortega

**Affiliations:** 1 Laboratorio de Inmunología Experimental, Subdirección de Medicina Experimental, Instituto Nacional de Pediatría, CDMX, México, México; 2 División Académica de Ciencias Agropecuarias, Universidad Juárez Autónoma de Tabasco, Tabasco, México; 3 Bioterio, Subdirección de Medicina Experimental, Instituto Nacional de Pediatría, CDMX, México, México; 4 Departamento de Medicina, Cirugía y Zootecnia para Équidos, FMVZ, UNAM, CDMX, México, México; 5 Programa de Doctorado en Ciencias de la Producción y de la Salud Animal, Facultad de Medicina Veterinaria y Zootecnia, Universidad Nacional Autónoma de México, México, México

**Keywords:** free-range chickens (*Gallus gallus domesticus*), genotyping, isolation, México, *Toxoplasma gondii*, virulence prediction

## Abstract

*Toxoplasma gondii* is a protozoan parasite that causes infection in birds and mammals (terrestrial and marine), both domestic and wild. The state of Tabasco has favourable climatic and ecological conditions for the replication and dissemination of this parasite. Therefore, the aim of this study was to isolate and genotype *T. gondii* from free-range chickens in this region of México by PCR-RFLP of 15 genetic markers. A total of 12 chickens were obtained from 7 municipalities. Serological survey by the modified agglutination test (MAT) of chicken serum samples revealed that 9 out of the 12 chickens (75%) tested had antibodies against *T. gondii* (titres ≥ 1:10). *Toxoplasma gondii* DNA was detected by PCR in tissues from 8 out of the 12 chickens. Twelve viable strains of *T. gondii* were isolated from the heart and brain samples of eight chickens by bioassay in mice. Genetic characterization of tachyzoite-derived DNA was performed using 10 multilocus RFLP-PCR genotyping markers (*SAG1, SAG2, SAG3, BTUB, GRA6, c22-8, c29-2, L358, PK1* and *Apico*) and five virulence-related markers (*CS3, ROP5, ROP16, ROP17* and *ROP18*). A total of 6 ToxoDB PCR-RFLP genotypes were identified, including #8 (also known as Type BrIII), #28, #38, and 3 new genotypes designated as #344, #345 and #346. Combination of *ROP18/ROP5* alleles were 1/3, 3/3 and 4/3. ToxoDB #344 and #345 genotypes also had a new allele at the *ROP5* locus. These results suggest high genetic diversity of *T. gondii* in southeastern México.

## Graphical abstract

**Figure:**
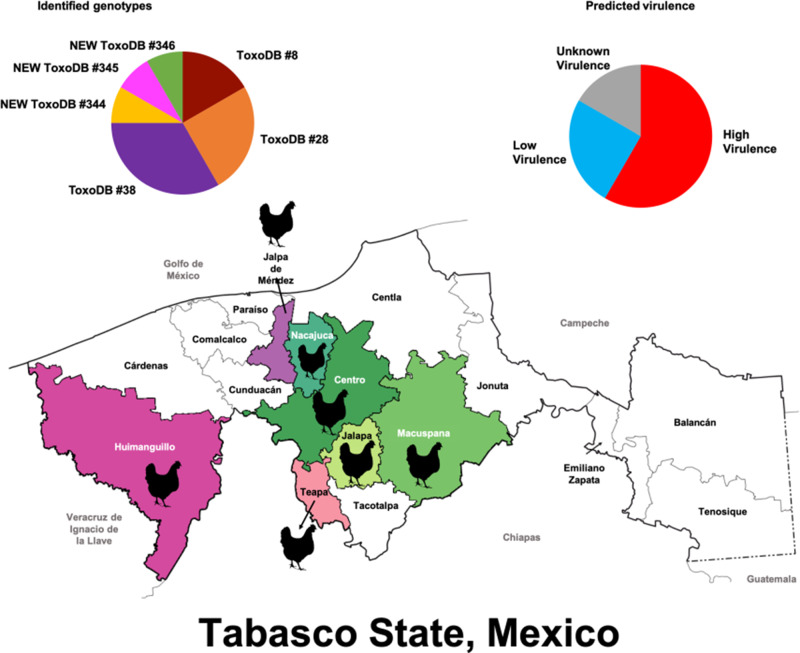

## Introduction

Toxoplasmosis is a zoonotic disease caused by *Toxoplasma gondii*, an obligate intracellular parasite that infects a wide variety of homeothermic animals, including humans. It can cause spontaneous abortions, stillbirths and congenital malformations in both humans and several animal species (Buxton, 1990; Tenter et al., 2000). It is estimated that approximately 30–50% of the world’s population is chronically infected by this protozoan, which is more common in warm and humid areas (Montoya and Liesenfeld, 2004; Dubey, 2010; Flegr et al., 2014). In México, approximately 44% of the human population is seropositive, with the Gulf and Pacific coasts being the southeastern regions with the highest seroprevalence of *T. gondii* infection (Caballero-Ortega et al., 2012). The Mexican southeast is a tropical region made up of warm-humid states such as Quintana Roo, Yucatán, Campeche, Chiapas and Tabasco, where the climatic and biotic conditions required for the perpetuation of *T. gondii* are ideal, along with the presence of wild felines, which benefit the spread and high genetic variability of the parasite (Comisión Nacional para el Conocimiento y Uso de la Biodiversidad (CONABIO) y Gobierno del Estado de Tabasco, 2021). Sentinel animals such as stray dogs, feral cats and free-range chickens are good indicators of the presence of this protozoan in the environment because they can consume water and food contaminated with *T. gondii* oocysts. Furthermore, most of the *T. gondii* isolates and genotypes described in the literature have been obtained from these animal species (Shwab et al., 2014; Calero-Bernal et al., 2022).

In addition to archetypal genotypes I, II and III, 343 different genotypes obtained from various animal species worldwide, including humans, are grouped into 6 different clades (Lorenzi et al., 2016; Gennari et al., 2024). Therefore, in areas with a high population density of definitive hosts (wild, feral or domestic cats), a wide variety of genotypes is expected due to sexual genetic recombination. We looked for the circulating genotypes of this parasite in the southeastern region of México because its genetic characteristics can directly influence the clinical presentation and severity of toxoplasmosis. We obtained *T. gondii* isolates (ToxoDB #3, #8, #10, #28, #38, #116, #164, #182, #225 genotypes) from feral cats from Quintana Roo, stray dogs from Chiapas and stray dogs and free-range chickens from Campeche, as well as several partial genotypes, mixed infections and the existence of up to 18 possible new genotypes of this parasite (Valenzuela-Moreno et al., 2019, 2020; Rico-Torres et al., 2024). These results suggest a considerable presence of different genotypes of *T. gondii*, including nonarchetypal strains that could be endemic to this region, and high infective pressure to which several intermediate hosts, including humans, are exposed. In addition to the classic genotyping panel, we have reported the genetic characterization of *CS3, ROP16, ROP17, ROP18* and *ROP5* markers that help predict the virulence of this parasite in mice. These loci play important roles in the parasite’s phenotype in murine models, as they modulate the host immune response through the ability to phosphorylate and activate transcription factors during invasion (Weilhammer and Rasley, 2011; Zhang et al., 2019). In particular, *ROP18*/*ROP5* allele combinations are highly predictive of virulence in a murine model (Gazzinelli et al., 2014; Shwab et al., 2016).

Continuing the genetic mapping of this parasite, we sampled free-range chickens from Tabasco to isolate and genotype *T. gondii* by Mn-PCR-RFLP, using 10 classical polymorphic markers and five specific genetic markers to predict its virulence in a murine model. Tabasco is located in the southeastern México that borders the Gulf of México, Veracruz, Chiapas, Campeche and Guatemala (Comisión Nacional para el Conocimiento y Uso de la Biodiversidad (CONABIO) y Gobierno del Estado de Tabasco, 2021), and to date there are no reports of *T. gondii* genotyping in this state.

## Materials and methods

### Tissue samples from free-range chickens

Twelve chickens were randomly sampled in the municipalities of Centro, Huimanguillo, Jalapa, Jalpa de Méndez, Macuspana, Nacajuca and Teapa in the State of Tabasco, México. The animals were euthanized with an intracelomic overdose of sodium pentobarbital (390 mg/kg, Dolethal^®^, Vetoquinol, France) following the guidelines of the Mexican Official Standard NOM-033-ZOO-1995. After confirming the loss of muscle activity and respiratory and cardiac arrest, necropsies were performed and representative sections of tissues (brain, lung, heart, liver and striated muscle) were collected and immersed in 10% buffered formalin (Schuneman and Constantino, 2002). Half of the heart and brain were kept at 4 °C and used for parasite isolation via a mouse bioassay.

### Serology

To determine the serologic status of the free-range chickens, the modified agglutination test (MAT) was standardized according to the methodology described by Dubey and Desmonts (1987) with slight modifications. *Toxoplasma gondii* GT1 tachyzoites were proliferated in NIH/3T3 mouse fibroblasts (ATCC CRL-1658) in 24-well plates (Costar, cat. 3524, Corning, NY, USA) and subsequently fixed in 6% formalin for 72 h, after which 0.2% Evans blue was added to the alkaline buffer. Sera from all chickens were diluted 1:5 in filtered PBS and serially diluted to 1:40. The MAT assay was carried out twice. Negative and positive serum controls were included in each plate (sera from naive and inoculated with Me49 strain mice, respectively). The sediment at the bottom of the plate was considered negative, and samples without sediment were considered positive.

### Biassay and isolation of *T. gondii*

Heart and brain samples from each chicken were digested with pepsin to purify and concentrate the *T. gondii* tissue cysts following the methodology described by Dubey (1998). Briefly, the tissues were cut into small pieces, macerated in a mortar with saline solution and digested with acidic pepsin solution in a water bath at 37 °C for 1 h. The homogenate was neutralized with 1.2% sodium bicarbonate (pH 8.3) and then suspended in saline solution with 1000 IU of penicillin and 100 μg /mL streptomycin (Sigma-Merck KGaA, Darmstadt, Germany). Finally, 1 mL of the homogenate from each tissue sample (heart and brain) was aseptically inoculated intraperitoneally into two BALB/c mice (one for each tissue). One week after inoculation, the peritoneal lavage of all of the mice was performed with 2 mL of sterile PBS to obtain tachyzoites. The obtained lavages were seeded on NIH/3T3 mouse fibroblasts (ATCC CRL-1658) in 24-well plates. After 3–7 days, the supernatant was transferred to NIH/373 cells growing at 70% confluency in 25 cm^2^ flasks (Falcon, cat. 353014, Corning, NY, USA), cultured in DMEM (ATCC, Washington, DC, USA) supplemented with 10% calf bovine serum (ATCC, Washington, DC, USA) and 1× antibiotic–antimycotic mixture (Gibco, MA, USA), and monitored daily for the appearance of tachyzoites. All of the isolated parasites were proliferated in cell culture and cryopreserved in 90% calf bovine serum (ATCC cat. 30-2020) and 10% dimethyl sulfoxide (DMSO, Sigma-Merck KGaA, cat. D2650, Darmstadt, Germany) in liquid nitrogen at −196 °C until use.

### DNA extraction

The genomic DNA from free tachyzoites collected from cell culture as well as from chicken tissues used to obtain *T. gondii* isolates was extracted using the Qiagen Gentra® Puregene® Tissue Kit (Hilden, Germany) following the manufacturer’s instructions and as previously reported (Cedillo-Peláez, 2009). DNA was quantified with a spectrophotometer (Thermo Scientific Nanodrop™ 1000, MA, USA) and stored at −20 °C until use.

### *Toxoplasma gondii* PCR, genotyping and parasite load

The DNA of all of the viable parasite-like isolates was analysed for the noncoding repetitive sequence 529RE of *T. gondii* by convectional PCR using previously described primers (Homan et al., 2000). Positive *T. gondii* isolates were processed by multilocus-nested PCR-RFLP (Mn-PCR-RFLP), which amplified 10 polymorphic markers (*SAG1, 5’3’ SAG2*, Alt. *SAG2, SAG3, BTUB, GRA6, c22-8, c29-2, L358, PK1* and *APICO*) to determine the genotype of each isolate and five additional polymorphic markers that predict its virulence in the murine model (*CS3, ROP5, ROP16, ROP17* and *ROP18*) (Pena et al., 2008; Su et al., 2010; Shwab et al., 2016). All of the molecular assays were carried out with AmpliTaq Gold^TM^ polymerase (Thermo Fisher Scientific, cat. 4311806, MA, USA) as previously described (Su et al., 2010; Rico-Torres et al., 2022). All of the assays included sterile water as a negative control and DNA from reference strains of Types I, II and III (RH, Me49 and VEG, *Toxoplasma gondii* ATCC^®^ 50611^TM^ and 50861^TM^) as positive controls. Additional negative and positive reamplification controls were included during nested assays to rule out cross-contamination. The PCR products were resolved on 1.5% TBE-agarose gels stained with ethidium bromide (EtBr) and then visualized and digitalized under UV illumination (Bio-DocIt, UVP™). RFLP assays were performed with restriction enzymes from New England Biolabs and Thermo Scientific (*Fsp*BI) using 3.5 U of each enzyme as previously described (Pena et al., 2008; Su et al., 2010; Shwab et al., 2016). The products obtained by RFLP were visualized on 3% TBE-agarose gels, stained with EtBr and digitalized. Restriction fragments that showed a different pattern than those of the reference strains (*ROP5* and *ROP17*) were electrophoresed on a 4–15% polyacrylamide gel stained with EtBr to improve the resolution. Parasite load was determined in chicken tissues by TaqMan-based quantitative PCR assays using the *T. gondii B1* gene as the genetic target. Standard curves were created by spiking between 50 and 5 × 10^6^ tachyzoites of the RH strain into brain and heart samples from uninfected BALB/c mice (Kompalic-Cristo et al., 2007; Cedillo-Peláez et al., 2011; Vargas-Villavicencio et al., 2016). Each sample was analysed in triplicate using a 48-well StepOne thermocycler (Thermo Fisher Scientific, MA, USA). The number of tachyzoites per microgram of tissue was calculated by interpolating the threshold cycle (Ct) value of each tissue sample within the standard curve and the fluorescence data were collected at the end of the PCR process using Step One v2.3 software to obtain the linear regression coefficient (*R*^2^) and amplification efficiency (Eff% ± 10%).

### Sequencing

The samples that presented a different RFLP pattern than the RH, Me49 or VEG reference strains were amplified again from the original DNA of each *T. gondii* isolate using internal primers. The amplicons obtained were sequenced both forward and reverse at the Instituto de Biología-UNAM, México, using standard Sanger methodology, with a 3730xl 96-capillary DNA Analyzer (Thermo Fisher Scientific, MA, USA). Chromatograms were analysed with SnapGene viewer® v 5.2, and the overall Phred quality was above 30.

### Phylogenetic network

To characterize the evolutionary relationships between the *T. gondii* isolates identified in this study and the representative strains of each haplogroup, a phylogenetic network was constructed. The genotyping marker data were analysed using SplitsTree4 software (Huson, 1998; Huson and Bryant, 2006). To determine the phylogenetic relationships, representative strains of *T. gondii* from each haplogroup were included: RH, GT1 (1); Me49, PTG, PRU (2); VEG, CTG (3); MAS (4); GUY-RUB (5); FOU, Wiktor (6); CAST (7); TgCtBr05 (8); P89 (9); GUY-VAND (10); Cougar (11); Type12, Type 12-X-A (12); Chinese1 (13); GAB2–2007-GAL-DOM2 (14); and TgCtCo05 (15), as well as Mexican strains found in different parts of México, including those isolated in this study.

## Results

**Figure 1.**
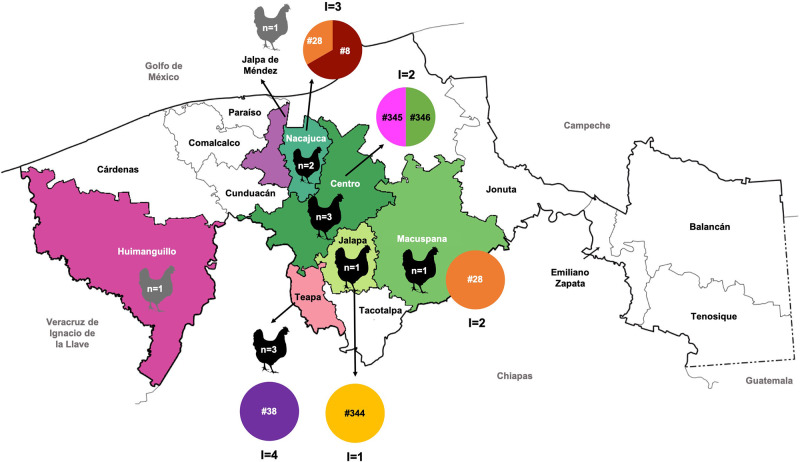
Sampling of chickens and genotype of *Toxoplasma gondii* isolates in the state of Tabasco, México. The municipalities from which biological samples of free-range chickens were obtained are shown. Grey chickens show individuals from which no isolates were obtained. n: number of chickens included per municipality. I: isolates obtained from chickens included in that municipality.

Twelve free-range chickens were obtained from 7 municipalities of Tabasco, México (Figure 1). All of the captured animals were adults and ranged from 6 months to 5 years old. During *post mortem* analyses, no lesions compatible with acute or chronic *T. gondii* infection were detected. Nine out of 12 chickens were positive for *T. gondii* according to the MAT test (titres from 1:10 to 1:40; Table 1).

**Table 1.** PCR-RFLP genotypes of *Toxoplasma gondii* isolates obtained from free-range chickens in the state of Tabasco, México

ID	Municipality	MAT	Isolate^a^	Tissue of isolation	*B1* qPCR^b^	Tachyzoites per mg tissue	Genetic markers											ToxoDB PCR-RFLP genotype
							*SAG1*	*5ʹ3’ SAG2*	*Alt. SAG2*	*SAG3*	*BTUB*	*GRA6*	*c22 − 8*	*c29 − 2*	*L358*	*PK1*	*APICO*	
Chicken 1	Macuspana	1:40	TgCkMxTab1a	Heart	+	11 583	I	I	I	I	I	I	II	I	III	I	III	#28
			TgCkMxTab1b	Brain	+	55	I	I	I	I	I	I	II	I	III	I	III	#28
Chicken 2	Nacajuca	Neg	TgCkMxTab2	Heart	+	<50	I	I	I	I	I	I	II	I	III	I	III	#28
Chicken 3	Nacajuca	1:20	TgCkMxTab3a	Heart	+	4503	I	III	III	III	III	III	II	III	III	III	III	#8 (BrIII)
			TgCkMxTab3b	Brain	+	920	I	III	III	III	III	III	II	III	III	III	III	#8 (BrIII)
Chicken 4	Teapa	1:40	TgCkMxTab4a	Heart	+	374	I	III	III	III	I	I	I	III	I	I	III	#38
			TgCkMxTab4b	Brain	+	1579	I	III	III	III	I	I	I	III	I	I	III	#38
Chicken 5	Teapa	1:40	TgCkMxTab5a	Heart	+	18 026	I	III	III	III	I	I	I	III	I	I	III	#38
			TgCkMxTab5b	Brain	+	14 581	I	III	III	III	I	I	I	III	I	I	III	#38
Chicken 6	Jalapa	1:40	TgCkMxTab6	Heart	+	2024	I	III	III	III	I	I	III	III	III	I	I	NEW #344
Chicken 7	Centro	1:40	TgCkMxTab7	Brain	+	1052	I	III	III	III	III	III	II	III	III	III	I	NEW #345
Chicken 8	Centro	1:10	TgCkMxTab8	Heart	+	2301	I	I	I	III	I	III	II	III	III	III	III	NEW #346

a *Toxoplasma gondii* strains were designated ‘a’ and ‘b’ when two isolates were obtained from the same chicken. ^b^A 62 bp fragment of the *B1* gene of *T. gondii* was detected in chicken tissues by qualitative PCR.

### Isolates, molecular identification and parasite load

**Figure 2.**
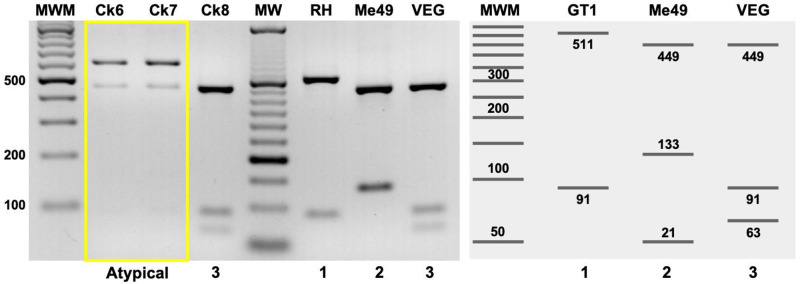
New *ROP5* allele identified in *Toxoplasma gondii* isolates from chickens in México. The restriction patterns of the Ck6 and Ck7 isolates (TgCkMxTab6 and TgCkMxTab7, respectively) were different from those of the reference strains RH, me49 and VEG, Types I, II and III, respectively (yellow box). The restriction products were resolved on 3% agarose gels stained with EtBr. MWM: molecular weight marker of 100 (agarose) and 50 (*in silico*) bp. Virtual digestion of the *ROP5* locus sequences of the archetypal strains GT1, Me49 and VEG, available at www.Toxodb.Org. Virtual digestion was performed with the benchling digestion tool (www.Benchling.Com). The image was converted to negative using GIMP v2.8 software.

Twelve viable isolates were obtained from the tissues of 8 chickens. One isolate was obtained from chickens 2, 6, 7 and 8, while 2 isolates each (one from the heart and one from the brain) were obtained from chickens 1, 3, 4 and 5 (Table 1). The four chickens from which no heart nor brain isolate was obtained were also negative for the presence of *T. gondii* DNA by PCR (data not shown). All of the isolates obtained were positive for the *T. gondii* marker 529RE by conventional PCR and were included in the multilocus nested PCR to obtain their genotype, as well as the genetic characterization of their virulence markers. Among the 12 *T. gondii* isolates, six different ToxoDB genotypes were identified: two #8 Type BrIII, three #28, four #38 and three new genotypes that were identified as #344, #345 and #346. The parasite load was determined in the tissues of origin and was equivalent to < 50 and more than 18 000 tachyzoites/mg of tissue (Table 1). The virulence marker *CS3* showed Type I and III alleles, whereas in *ROP16, ROP17, ROP18* and *ROP5*, the Type 1 alleles were more frequent than Type 3 and these more frequent than Type 4. Interestingly, in the TgCkMxTab6 and TgCkMxTab7 isolates, the *ROP5* restriction pattern was different from that of the archetypal Type I, II and III strains (RH, Me49 and VEG, respectively), as well as five other restriction patterns previously reported for this locus. Three *ROP18*/*ROP5* virulence patterns were identified in the 12 *T. gondii* isolates: three 4/3 (TgCkMxTab1a, 1b and 2), four 1/3 (TgCkMxTab4a, 4b, 5a and 5b) and three 3/3 (TgCkMxTab3a, 3b and 8). Two restriction patterns (TgCkMxTab6 and 7) could not be associated with virulence because of the new RFLP pattern at the *ROP5* locus (Figure 2 and Table 2). The *ROP17* RFLP digestion products of isolates TgCkMxTab7 and TgCkMxTab8 had a restriction pattern compatible with allele 4, which was confirmed by electrophoresis of the digestion products on polyacrylamide gels (Suppl Figure 1).

**Table 2.** Virulence multilocus PCR-RFLP typing *Toxoplasma gondii* isolates from free-range chickens in the state of Tabasco, México

Isolate ID	ToxoDB PCR-RFLP	Virulence genetic markers					Virulence prediction in mouse bioassay ^+^
		*CS3*	ROP16*	*ROP17*^	*ROP18*	*ROP5*	
RH	#10	I	1	1	1	1	High virulence
Me49	#1	II	2	2	2	2	Low virulence
VEG	#2	III	1	2	3	3	Low virulence
**Present study**							
TgCkMxTab1a	#28	I	1	1	4	3	High virulence
TgCkMxTab1b	#28	I	1	1	4	3	High virulence
TgCkMxTab2	#28	I	1	1	4	3	High virulence
TgCkMxTab3a	#8 BrIII	III	1	1	3	3	Low virulence
TgCkMxTab3b	#8 BrIII	III	1	1	3	3	Low virulence
TgCkMxTab4a	#38	I	1	1	1	3	High virulence
TgCkMxTab4b	#38	I	1	1	1	3	High virulence
TgCkMxTab5a	#38	I	1	1	1	3	High virulence
TgCkMxTab5b	#38	I	1	1	1	3	High virulence
TgCkMxTab6	#344	III	1	1	3	**Atypical**	**Unknown**º
TgCkMxTab7	#345	III	1	4	3	**Atypical**	**Unknown**º
TgCkMxTab8	#346	III	1	4	3	3	Low virulence

* Strains type I and III have allele 1. ^Strains type II and III have allele 2. + According to Shwab et al. (2016). ºThere is no virulence profile for this combination.

### Sequencing

The *ROP17* locus of isolates TgCkMxTab7 and TgCkMxTab8 presented a different restriction pattern from that of the archetypal alleles (RH type 1, Me49 and VEG type 2). Therefore, they were amplified again using the external primers and then sequenced. The obtained sequence was subjected to virtual digestion (www.benchling.com), which replicated the restriction pattern of two known *T. gondii* strains with type 4 alleles of *ROP17,* MAS and TgCatBr5. The sequences obtained from these two isolates were subsequently aligned and compared with the *ROP17* sequences of the archetypal strains GT1, Me49 and VEG and of the nonarchetypal strains MAS and TgCatBr5. The two *ROP17* sequences obtained from *T. gondii* strains in chickens had 100% identity with the TgCatBr5 sequence and 99.9% (796/797) identity with the MAS *T. gondii* strain, which has a SNP at position 77 of the alignment (Suppl Figure 2). The obtained and analysed sequences were deposited in GenBank® with accession numbers PP983038 and PP983039.

### Phylogenetic network

**Figure 3.**
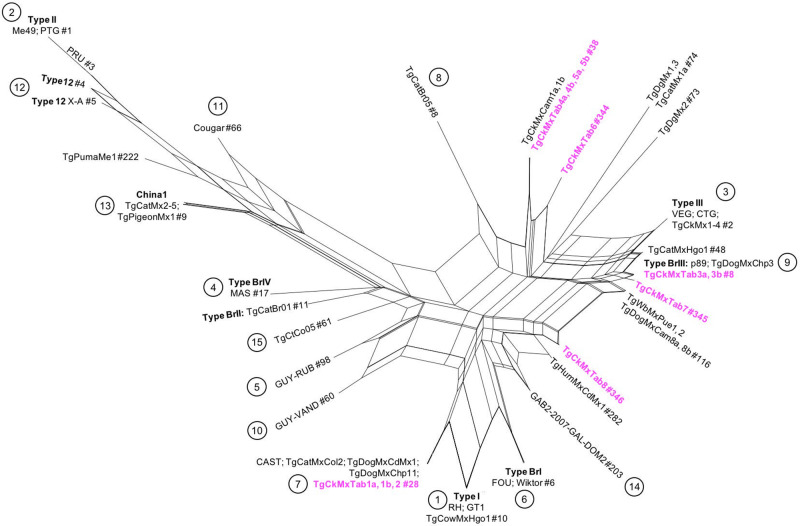
Phylogenetic network of *Toxoplasma gondii* isolates obtained from chickens in Tabasco, México. the network was built using PCR-RFLP data of the isolates obtained from Tabasco along with Mexican and reference strains of each haplogroup (circled in black). The genotype IDs of the *T. gondii* strains described in this study are highlighted in magenta.

The *Toxoplasma gondii* isolates obtained in this study were grouped mainly into Type I and Type III branches or close to them. The isolates TgCkMxTab1a, 1b and 2 shared a branch with other isolates of *T. gondii* genotype #28 obtained in México and with the CAST strain, which is representative of haplogroup 7. TgCkMxTab3a and TgCkMxTab3b were placed on the same branch as a *T. gondii* isolate obtained from a dog from the State of Chiapas (genotype #8) and P89 strain in haplogroup 9, along with another Mexican isolate (TgCatMxHgo1, genotype #48) in the branch of genotypes related to Type III strains. The TgCkMxTab4a, 4b, 5a and 5b isolates clustered with *T. gondii* isolates from the State of Campeche near the TgCatBr05 branch and between haplogroups 3 and 8. Finally, the TgCkMxTab6, 7 and 8 isolates (new genotypes) do not share a branch with any included strain but are close to haplogroups 3, 9 and 14, respectively (Figure 3).

## Discussion

México is located in a climatic transition region between North America and Central and South America. Tabasco is a southeastern state with a tropical climate and favourable conditions for the perpetuation and dissemination of *T. gondii*. Therefore, it is interesting to determine if the circulating genotypes of this region are the same as those described in neighbouring states and other tropical regions of the continent. Free-range chickens are ideal sentinel animals for ground contamination because they feed on soil organic matter and can come in contact with oocysts released by felines (Dubey et al., 2007; Shwab et al., 2014; Kurth et al., 2021). The high frequency of anti-*T. gondii* antibodies in chickens was 75% (9/12) is consistent with the frequency reported in stray dogs from Chiapas and Campeche and in humans from Yucatan Peninsula, which suggests that peridomestic animals are good indicators of the infective pressure of *T. gondii* in the environment (Caballero-Ortega et al., 2012; Valenzuela-Moreno et al., 2020; Rico-Torres et al., 2024).

Twelve isolates were obtained from eight chickens that resulted in 6 different genotypes of *T. gondii*, three that had already been reported in México and other countries on the American continent and three previously unpublished genotypes. The ToxoDB #8 genotype (Type BrIII) was first isolated in México from a stray dog from Chiapas, and it is widely spread in Brazil and other American countries, such as the USA and Venezuela (Pena et al., 2008; Shwab et al., 2014; Valenzuela-Moreno et al., 2020). This is the second time that ToxoDB #38 has been isolated in México, and it has otherwise only previously been reported in Colombia, but it could be distributed throughout the tropical regions of southern México and Central and South America (Rajendran et al., 2012; Rico-Torres et al., 2024). The ToxoDB #28 genotype has been isolated several times in states on the Pacific coast and central and southeastern México; thus, it could be a regional genotype similar to BrI-BrIV and China I of Brazil and China, respectively (Shwab et al., 2014; Dardé et al., 2020; Rico-Torres et al., 2023).

The three new genotypes identified in this study are ToxoDB #344, #345 and #346. The first is a recombinant strain I-III with an even number of Type I and III alleles, whereas the remaining two genotypes are recombinant strains I–II–III with a majority of Type III alleles. Notably, genotype #345 is almost identical to genotype #8, except for the Type I allele at the *Apico* locus instead of Type III.

According to Shwab et al. (2016), there is a correlation between the combination of *ROP18/ROP5* genetic markers and the outcome of infection in a murine model. At least six genetic profiles have been identified that predict high virulence (mortality > 80%), two with intermediate virulence (31–79%) and five with low virulence (<30%). Among the *T. gondii* isolates obtained in Tabasco, two with high virulence (4/3 and 1/3) and one with low virulence (3/3) were identified. Additionally, an atypical profile could not be associated with virulence because the *ROP5* restriction pattern was different from those previously reported (Shwab et al., 2016). The resulting restriction pattern is similar to that of a type III (faint) allele, but most of the product remained undigested (Figure 2). The *ROP5* locus is composed of three paralogous genes arranged in tandem (*ROP5a, ROP5b* and *ROP5c*). The primers designed for the *ROP5* locus amplify these three paralogous genes; therefore, the isolates TgCkMxTab6 and TgCkMxTab7 may have some copies of an *ROP5* isoform with restriction sites for the *Fsp*BI enzyme that shows a faint Type III pattern, and the other paralogues may have lost their restriction sites, leaving them uncut (Reese et al., 2011; Shwab et al., 2016). Furthermore, this is the second time we found a different restriction pattern for this particular locus, the first being a recombinant locus 1/3 identified in two *T. gondii* isolates obtained from a stray dog from Campeche, so it is important to phenotype these strains and determine the degree of virulence of these new alleles (Rico-Torres et al., 2024). For the *ROP18* locus, three type 4 and four type 1 alleles were obtained from the ToxoDB #28 and #38 isolates, respectively. Both alleles are associated with high virulence in mice, resulting in up to 100% mortality. Finally, two previously unreported *T. gondii* strains with allele 4 at the *ROP17* locus were obtained. Although this gene is not directly related to the phenotype of *T. gondii*, strains carrying this allele cause mortality close to 80% in mice; thus, the resulting protein could be more efficient in helping the *ROP18/ROP5* complex and *GRA7* to phosphorylate the IRGs (Shwab et al., 2016). The expanded panel of virulence typing markers allowed us to differentiate the ToxoDB #8 isolates obtained in this study from those typed from the state of Chiapas, the latter with a type 4 allele in *ROP17*, such as the P89 strain, whereas the strains isolated from Tabasco have a type 1 allele that makes them identical to several Brazilian strains (Shwab, 2015; Valenzuela-Moreno et al., 2020). The virulence prediction should be taken with caution, as it only considers the ROP18/ROP5 combination and does not include other proteins involved in this complex, such as ROP17, GRA7, and ROP39, which may enhance or attenuate the effects of ROP18/ROP5 combination in inactivating IRGs (Murillo-Léon et al., 2024).

The constructed phylogenetic network suggests that *T. gondii* has high rates of recombination, in which frequent genetic exchange has resulted in a variety of recombinant strains and the proliferation of what appears to be a successful genotype, ToxoDB #28 (Morrison, 2005; Pena et al., 2008). The ToxoDB #28 strains clustered in the main branch of Type I (haplogroup 1) and with Type I-related strains such as FOU and Wiktor (haplogroup 6; BrI type) and CAST (haplogroup 7), which are known to be virulent in mice, suggesting that genotype #28 could be virulent in mice (as predicted by the *ROP18*/*ROP5* profile). The ToxoDB #8 and #345 genotypes were grouped in the Type III strain branch (haplogroups 3 and 9) near other Mexican isolates, with a predominance of the Type III background. Finally, the ToxoDB #38 strains clustered with the isolates reported in Campeche, whereas genotypes #344 and #346 did not cluster with any reported genotype since this is the first time they have been described and they remained in individual branches close to other Mexican isolates. None of the genotypes described in this study clustered near Type II archetypal or Type II-related strains. The discovery of three new *T. gondii* genotypes carrying atypical alleles in virulence-related genes in laboratory mice raises many questions about the impact they may have on humans and both wild and domestic animals in the state of Tabasco. Phylogenetically, genotype #345 is located on the same branch as genotype #8 (Type BrIII), which is considered avirulent in mice (Pena et al., 2008). This suggests that it may pose a low risk to other species in the region. In contrast, genotype #346 does not share a branch with any of the included strains but is close to genotype #282, which was isolated from a fatal case of congenital toxoplasmosis; therefore, this genotype could be virulent in such cases (Rico-Torres et al., 2018, 2023). Finally, genotype #344 is also located on its own branch but is adjacent to ToxoDB #38, which has been predicted to be highly virulent due to the *ROP18/ROP5* allele combination. However, its impact on animals and humans in the region remains uncertain. Tabasco is home of species considered highly susceptible to toxoplasmosis, such as the howler monkey (*Alouatta palliata* and *Alouatta pigra*) and the spider monkey (*Ateles geoffroyi*), which are listed as endangered by the Mexican Official Standard NOM-059-SEMARNAT-2010 and could be at risk of developing clinical problems and a fatal outcome if infected with these strains (Ceballos and Oliva, 2005; Dubey, 2010).

In conclusion, the *T. gondii* isolates TgCkMxTab6, TgCkMxTab7 and TgCkMxTab8 have the new genotypes ToxoDB#344, #345 and #346, respectively. In addition, we found *T. gondii* genotypes #8, #28 and #38, which have been previously described in other neighbouring states, suggesting that they are circulating in the tropical areas of southeastern México. Genotype #28 is becoming a frequent finding in different states of México. The ToxoDB#344 and #345 genotypes had an atypical allele at the *ROP5* locus, and this could be the ninth variant found for this paralogous gene. The combination of *ROP18/ROP5* alleles predicts high virulence in most of the *T. gondii* isolates obtained in the present study. However, this prediction must be confirmed by mouse bioassay. This study provides evidence of endemic *T. gondii* strains found in intermediate hosts to which other hosts, including humans, may be exposed.

## Supporting information

Valenzuela-Moreno et al. supplementary material 1 Valenzuela-Moreno et al. supplementary material

Valenzuela-Moreno et al. supplementary material 2 Valenzuela-Moreno et al. supplementary material

Valenzuela-Moreno et al. supplementary material 3 Valenzuela-Moreno et al. supplementary material

## Data Availability

Reference strain sequences of GT1, Me49 and VEG (*ROP5* and *ROP17*) are available at: www.toxodb.org. TgCkMxTab7-ROP17 PP983038 and TgCkMxTab8-ROP17 PP983039 are available at: https://www.ncbi.nlm.nih.gov/genbank/. ToxoDB genotypes are available at: http://web.utk.edu/~csu1/ListToxoDB-PCR-RFLPgenotypes.html.

## Supplementary material

The supplementary material for this article can be found at https://doi.org/10.1017/S0031182025100401.
