# Supplementary material for: New genetic variants of Toxoplasma gondii isolates and a novel ROP5 allele in free-range chickens from Tabasco, México
Source: Parasitology. 2025 Jul 1;152(10):1083–91. doi: 10.1017/S0031182025100401 (PMC12644966; doi:10.1017/S0031182025100401)
Supplement: Valenzuela-Moreno et al. supplementary material 1 — Valenzuela-Moreno et al. supplementary material [file S0031182025100401sup001.docx]

**Supplementary Figure 1.** Representative RFLP patterns for the *Toxoplasma gondii* *ROP17* allele. A) Type 4 alleles were found in the *T. gondii* isolates TgCkMxTab7 and TgCkMxTab8 (Ck7 and Ck8, respectively) and are highlighted in a red box. RH, Me49 and VEG are Type I, II and III reference strains, respectively. B) Virtual digestion of the *ROP17* locus of the archetypal (GT1, Me49 and VEG) and nonarchetypal strains (Cougar and MAS) bearing atypical alleles (3 and 4) along with the sequences obtained from Ck7 and Ck8 (red box). *In silico* digestion was performed with the Benchling digestion tool (www.benchling.com). The *ROP17* genotype of each strain is indicated with Arabic numerals below. C) Confirmation of type 4 alleles in the RFLP products of the *T. gondii* isolates TgCkMxTab7 and TgCkMxTab8 was performed in 4–15% polyacrylamide gels and stained with EtBr. MWM: Molecular weight marker of 50 bp. The image was converted to negative using GIMP v2.8 software.

**Supplementary Figure 2.** Multiple sequence alignment of the *ROP17* locus of *Toxoplasma gondii* isolates from Tabasco, México. The sequences obtained from the isolates TgCkMxTab7 (ToxoDB #344) and TgCkMxTab8 (ToxoDB #345) were aligned with the *ROP17* reference strain sequences GT1, Me49 and VEG (Types I, II and III, respectively) and strains that bear a type 4 allele (MAS and TgCatBr5). The portions of the sequences that are not shown in the alignment are conserved regions among the aligned strains. Position 77 of the alignment is highlighted in a red box, where the strains TgCatBr5, TgCkMxTab7 and TgCkMxTab8 have a SNP compared with the MAS strain (G/A). The alignment was built using BioEdit® v5.0.6 software, and sequences from chromosome VIIb of all included strains of *T. gondii* were downloaded from [www.toxodb.org](http://www.toxodb.org).
